# Mental health and self-harm/suicide risk screening at prison entry over 12 months in a total population sample in New South Wales, Australia

**DOI:** 10.1177/00048674251336031

**Published:** 2025-04-28

**Authors:** Carey Marr, Christie Browne, Dylan Ngui, Reem Zeki, Emma Woods, Kimberlie Dean

**Affiliations:** 1 Discipline of Psychiatry and Mental Health, School of Clinical Medicine, University of New South Wales, Sydney, NSW, Australia; 2 Justice Health and Forensic Mental Health Network, Sydney, NSW, Australia; 3 School of Medicine and Public Health, University of Newcastle, Newcastle, NSW, Australia; 4 Medical School, University of Exeter, Exeter, UK

**Keywords:** Prison, mental health, self-harm, suicide, mental illness, screening

## Abstract

**Introduction:**

International research has found high rates of mental illness and self-harm/suicide among people in prison. However, existing studies are often limited by their sample selection methodology, and many do not examine mental health at prison entry specifically. In addition, with smaller samples, previous studies have lacked statistical power to robustly examine and compare subgroups.

**Method:**

This study examines a total population sample of 9568 individuals entering public prisons in New South Wales, Australia over a 1-year period, and describes the prevalence of a range of mental health and self-harm/suicide risk indicators collected during routine health screening upon prison entry.

**Results:**

In total, 62% of prison entrants reported a lifetime mental health condition and 23% reported a serious mental illness. Lifetime self-harm (14%) and suicide attempts (12%) were also high. Women and First Nations people entering prison had higher prevalence of most mental health diagnoses and of previous self-harm and suicidal ideation/behaviour than men and non-First Nations people.

**Conclusion:**

These results establish the scale of mental health need and self-harm/suicide risk among people entering prison, particularly among women and First Nations people. There is a clear need for investment to adequately resource prison-based mental health services to meet the needs of prison entrants, but also in interventions to prevent those with significant mental health needs from entering prison in the first place. These strategies may include targeted and preventive approaches via mental health diversion and community-based mental health services.

## Introduction

Meta-analytic work has confirmed high rates of mental illness and self-harm/suicide among people in prison around the world (Fazel and Seewald, 2012). For example, across 109 samples from 24 countries, pooled prevalence rates of psychosis were found to be around 3.6% for men in prison and 3.9% for women in prison, and pooled prevalences of major depression to be around 10.2% for men and 14.1% for women (Fazel and Seewald, 2012). While less has been published on other mental health conditions, a growing evidence base indicates the same is true for other mental disorders, including post-traumatic stress disorder (PTSD; Favril et al., 2024). Substance use problems are also well known to be highly prevalent among people in prison (e.g. Fazel et al., 2017). Higher incidence and prevalence of suicide attempts and ideation of people in prison has also been found both internationally and in Australia (Butler et al., 2018; Mundt et al., 2024).

Furthermore, rates of mental illness have been found to be higher for women and ethnic minority groups in prison (Sirdifield et al., 2009). These findings are also supported by some Australian studies. For example, higher prevalence of mental disorders and self-harm or suicide behaviours have been found for women in prison in New South Wales (NSW) over a series of prison surveys (Browne et al., 2022a, 2022b; Butler et al., 2005; Larney et al., 2012). Similar findings exist regarding elevated rates of mental illness and self-harm or suicide behaviours for First Nations people in custody in Australia (e.g. Heffernan et al., 2012; Larney et al., 2012; Ogloff et al., 2017).

There is limited meta-analytic work examining the prevalence of mental health conditions and self-harm/suicide risk at prison entry. A large meta-analysis by Fazel and Seewald (2012) reported results from prison studies using diagnostic interviews; however, only 9 of the 81 included studies had these interviews conducted at prison entry. The prevalence of mental health conditions in prison is known to vary throughout the course of imprisonment, with some evidence showing that psychiatric symptoms may be elevated at prison entry and improve over time (Hassan et al., 2011; Walker et al., 2014). In addition, many studies examining the prison entry period suffer from other limitations, including limited sample size, low and differential response rates, and/or using non-representative convenience sampling. Large representative samples are necessary for undertaking key subgroup analyses. For example, women make up only 8% of the custodial population in Australia (Australian Bureau of Statistics, 2024). Such limitations reduce the ability of researchers to conduct sufficiently powered and reliable subgroup analyses to better understand the rates and risks of mental health, self-harm and suicide for people prison and thus inform the development of effective health service interventions.

This study aims to describe the prevalence of a range of mental health and self-harm/suicide risk indicators collected during routine health screening upon public prison entry in NSW. Unlike many previous studies, this study takes a whole-of-population approach, reporting data collected for a study population of all individuals entering public prisons in NSW, ensuring that selection and attrition biases are avoided. This study also aims to examine differences in prevalence across two key subgroups: those defined by gender (men vs women) and First Nations identity (First Nations vs non-First Nations).

## Method

### Setting and study population

The data for this study were obtained from the *People in NSW Public Prisons: Health Status and Service Utilisation* project’s dataset held by Justice Health and Forensic Mental Health Network, the public health service tasked with providing health services to people in public prisons in the Australian state of NSW. This dataset includes routinely collected administrative data over a 1-year period (2 February 2021 to 31 January 2022). This includes data from the Reception Screening Assessment (RSA), a health assessment completed in public prisons in NSW.

In total, there were 9568 unique individuals entering prison during this time period, comprising 8219 men (85.9%) and 1347 women (14.1%; missing data on gender for two individuals). Around 12% of individuals (*n* = 1142) entered prison more than once during the period, most of which (87%) had two receptions. In total, there were 10,822 entries to prison, though only the first reception of each individual was included in the analyses in this paper. There were missing data, and all analyses were undertaken on a complete data basis. The amount of missing data can be found in Table 1 (column titled ‘*Total N*’). The variable with the most missing data was ‘*Ever engaged in self-harm*’, with data missing from 1.65% of the sample.

**Table 1.** Overview of prison-entry study population.

Variable	Total *N*	*n*	%
Demographic characteristics			
Median age	9568	*Median* *=* 34	*IQR* *=* 27-42
Male	9566	8219	85.9
First Nations	9508	3045	32.0
First time in custody	9485	2548	26.9
Mental health variables			
Any mental health condition reported	9479	5874	62.0
History of depression	9465	4463	47.2
History of anxiety	9458	3928	41.5
History of schizophrenia	9452	1287	13.6
History of bipolar or manic depression	9449	974	10.3
History of drug-induced psychosis	9449	614	6.5
History of PTSD or other trauma^a^	9439	749	7.9
Experienced mental illness symptoms in past month	9413	2204	23.4
Psychotic symptoms in the past month	9413	908	9.6
Serious mental illness^b^	9479	2173	22.9
Common mental disorder^c^	9479	4926	52.0
Mental health referral recommended	9568	3939	41.2
Mental health referral made	9567	5303	55.4
Suicide and self-harm variables			
Ever engaged in self-harm	9411	1336	14.2
Ever attempted suicide	9414	1135	12.1
Any suicidal ideation in past week	9419	679	7.2
Any self-harm in past 12 months	9435	611	6.5
Any suicide attempts in past 12 months	9435	471	5.0
Any self-harm or suicide attempts in past 12 months	9435	797	8.4
RIT referral recommended	9567	733	7.7
RIT referral made	9567	1020	10.7

Total study population = 9568.

a This variable was coded from ‘Other mental illness’ prompt.

b Includes individuals with a history of schizophrenia, psychosis, and/or bipolar disorder.

c Includes individuals with a history of depression and/or anxiety.

### Measures

Upon entry to prisons in NSW, all individuals complete an RSA, facilitated by a primary care nurse. The RSA is a comprehensive health screen which informs referrals to health services in prison and is typically completed with individuals within 24 hours of prison entry. In instances where a full assessment is not feasible due to the patient’s psychological or physical condition – such as uncooperative behaviour, confusion, violence, sedation or refusal of assessment – an RSA form must still be initiated by the primary care nurse and the reasons for any delay in completion or incomplete finalised assessment are noted. A record of any clinical observations is also captured in the RSA, allowing for the primary care nurse to report any observations indicative of a mental health condition (e.g. bizarre behaviour, agitation, apparent responding to hallucinations). For those who speak a language other than English or are not fluent in English, Health Care Interpreters are engaged where possible.

The mental health component of the RSA, called the Prison Mental Health Screening (PMHS) tool, screens for key mental health conditions and symptoms, as well as any history of mental health treatment and/or suicidal and self-harm behaviour including recent ideation. This tool is used in NSW and was developed and tested by the current research team (Browne et al., 2022a; Dean et al., 2024). A copy of the relevant questions from this tool can be found in Supplementary Materials. The screening tool is administered by generally trained nursing staff who complete the digital form during the assessment interview with each prison entrant. The tool utilises algorithms based on the responses to the screening items to aid clinical decision-making. Recommendations for referrals are then made to custodial mental health services or the Risk Intervention Team (RIT), a team responsible for assessing and managing incarcerated individuals who are at an increased risk of suicide or self-harming behaviour.

#### Mental health

For this study, self-reported histories of the following diagnoses were examined: depression, anxiety, schizophrenia, bipolar or manic depression and drug-induced psychosis. There was also an open-ended question asking about other mental health problems, from which we coded PTSD or other trauma (noting that this condition is thus likely underreported). Two mental health categories were created by combining specific items: *Serious mental illness*, which included a self-reported history of schizophrenia, psychosis or bipolar disorder, and *Common mental disorder*, which included participants who reported a history of depression, anxiety or both. Also examined was the presence of any key symptoms of a mental health condition within the past month, as well as any symptoms specifically associated with psychosis (hallucinations, paranoia, unusual beliefs and thought broadcasting) in the past month. Recommendations for referral to custodial mental health services was also examined, as well as actual referrals made.

#### Suicide and self-harm risk

The screening items related to suicide and self-harm risk examined in this study included a lifetime history of self-harm or suicide attempt, self-harm or suicide attempt within the past 12 months, and any suicidal ideation in the past week. Any suicidal ideation within the past week was determined by four screening questions regarding passive and active thoughts of suicide the past week: *Have you been thinking that you might be better off dead? Have you had any thoughts that life is not worth living? Have you had any thoughts about hurting or killing yourself? Have you had any thoughts about how you might end your life?*

Also examined was whether a recommendation for referral to the RIT was received by the screener as per the tool’s in-built algorithm, which generates a recommendation if an individual endorsed having engaged in a suicide attempt or self-harm in the past 4 weeks or indicated active suicidal ideation (thoughts of hurting or killing oneself or suicide plan within the last week).

### Statistical analysis

Data analysis was performed using SPSS Statistics 28 to examine the frequency of demographic, mental health, and suicide and self-harm variables within the overall study population. Chi-square tests were conducted to make comparisons of prevalence of all variables between (1) men and women, and (2) First Nations and non-First Nations people.

### Ethical approval

Ethics approval for this study was granted by the Justice Health and Forensic Mental Health Network Human Research Ethics Committee (Ref: G185/14), the NSW Aboriginal Health and Medical Research Council Ethics Committee (Ref: 1137/15) and the Corrective Services NSW Ethics Committee (Ref: D16/139081). A waiver of consent was granted, allowing for the inclusion of the routinely collected data from all prison entrants in this study.

## Results

An overview of demographic, clinical and self-harm/suicide characteristics of the prison-entry study population is presented in Table 1. In total, 85.9% of prison entrants were men and 32.0% identified as First Nations. The median age of prison entrants was 34 years (ranging from 18 to 86 years). The majority of prison entrants reported having been in prison before, with just 26.9% of people screened were entering custody for the first time.

In total, 62.0% reported a history of any mental health condition, with 23.4% experiencing symptoms of a mental health condition within the past month. The most common diagnoses reported were a history of depression (47.2%) or anxiety (41.5%). Around 14% of prison entrants reported history of schizophrenia, and 9.6% reported experiencing psychotic symptoms within the past month. When disorder types were grouped, just under a quarter (22.9%) had a serious mental illness and over half (52.0%) had a common mental disorder. Around 14% of prison entrants reported any history of self-harm, with 6.5% reporting self-harm in the past year. Past suicide attempts were slightly lower, with 12.1% reporting any attempt in the past, and 5.0% reporting an attempt within the past year. Over 7% reported suicidal ideation within the past week. Referrals to the prison mental health service were made for 55.4% of prison entrants and 10.7% were referred to RIT.

### Comparison of men and women

Table 2 outlines the comparison between men and women across all variables. A greater proportion of women reported being First Nations (38.4% vs 31.0% of men) and being in custody for the first time (31.6% vs 26.1% of men).

**Table 2.** Gender comparison.

Variable	Men	Women	χ^2^	*p*-value	ϕ
	*n* = 8219	*n* = 1347			
Demographic characteristics					
Under 30 years	2981 (36.3%)	552 (41.0%)	11.02	<0.001	–0.034
First Nations	2532 (31.0%)	513 (38.4%)	28.93	<0.001	–0.055
First time in custody	2124 (26.1%)	422 (31.6%)	17.78	<0.001	–0.043
Mental health variables					
Any mental health condition reported	4837 (59.4%)	1037 (77.6%)	161.40	<0.001	–0.131
History of depression	3582 (44.1%)	881 (66.0%)	222.12	<0.001	–0.153
History of anxiety	3122 (38.4%)	806 (60.5%)	228.87	<0.001	–0.156
History of schizophrenia	1111 (13.7%)	176 (13.2%)	0.21	0.650	0.005
History of bipolar or manic depression	783 (9.6%)	191 (14.4%)	27.35	<0.001	–0.054
History of drug-induced psychosis	551 (6.8%)	63 (4.7%)	7.91	0.005	0.029
History of PTSD or other trauma^a^	568 (7.0%)	181 (13.6%)	68.36	<0.001	–0.85
Experienced mental health symptoms in past month	1809 (22.3%)	395 (30.0%)	36.89	<0.001	–0.63
Psychotic symptoms in the past month	772 (9.5%)	136 (10.3%)	0.808	0.369	–0.009
Serious mental illness^b^	1839 (22.6%)	334 (25%)	3.77	0.052	–0.020
Common mental disorder^c^	3976 (48.8%)	950 (71.1%)	228.00	<0.001	–0.155
Mental health referral recommended	3128 (38.1%)	811 (60.2%)	234.42	<0.001	–0.157
Mental health referral made	4306 (52.4%)	997 (74.0%)	218.96	<0.001	–0.151
Suicide and self-harm variables					
Ever engaged in self-harm	989 (12.2%)	347 (26.1%)	180.65	<0.001	–0.139
Ever attempted suicide	930 (11.5%)	205 (15.5%)	17.04	<0.001	–0.043
Any suicidal ideation in past week	550 (6.8%)	129 (9.7%)	14.41	<0.001	–0.039
Any self-harm in past 12 months	448 (5.5%)	163 (12.3%)	85.54	<0.001	–0.095
Any suicide attempts in past 12 months	385 (4.8%)	86 (6.5%)	7.12	0.008	–0.027
Any self-harm or suicide attempts in past 12 months	617 (7.6%)	180 (13.5%)	51.91	<0.001	–0.074
RIT referral recommended	584 (7.1%)	149 (11.1%)	25.59	<0.001	–0.052
RIT referral made	800 (9.7%)	220 (16.3%)	52.88	<0.001	–0.074

*Note.* Degrees of freedom = 1. Totals ranged from 9411 to 9566 due to missing data.

a This variable was coded from ‘Other mental illness’ prompt. ^b^Includes individuals with a history of schizophrenia, psychosis, and/or bipolar disorder. ^c^Includes individuals with a history of depression and/or anxiety.

Several mental health variables were found to be reported more often by women than men, with the exception of a history of drug-induced psychosis, which had a higher prevalence for men (6.8%) than women (4.7%). The largest excesses for women compared to men were observed for ‘any’ mental health condition reported (77.6% vs 59.4%), a history of depression (66.0% vs 44.1%), a history of anxiety (60.5% vs 38.4%), and a mental health referral recommended (60.2% vs 38.1%) or made (74.0% vs 52.4%).

Women also showed statistically significantly higher prevalence of all self-harm and suicide risk variables. The largest effects were observed for ever having engaged in self-harm (26.1% vs 12.2%), self-harm in the past 12 months (12.3% vs 5.5%) and having a RIT referral recommended (11.1% vs 7.1%) or made (16.3% vs 9.7%).

### First Nations identity comparison

Table 3 outlines the comparison between First Nations and non-First Nations prison entrants across all variables. Around 45% of First Nations prison entrants were under 30, compared with 33.2% of non-First Nations prison entrants. A greater proportion of non-First Nations prison entrants were male (83.2% vs 87.3%) and reported being in custody for the first time (16.8% vs 31.6%).

**Table 3.** First Nations identity comparison.

Variable	First Nations	Non-First Nations	χ^2^	*p*-value	ϕ
	*n* = 3045	*n* = 6463			
Demographic characteristics					
Male	2532 (83.2%)	5638 (87.3%)	28.93	<0.001	-0.055
Under 30 years	1370 (45.0%)	2144 (33.2%)	124.08	<0.001	0.114
First time in custody	504 (16.8%)	2031 (31.6%)	229.71	<0.001	-0.156
Mental health variables					
Any mental health condition reported	2117 (70.5%)	3717 (58.0%)	139.49	<0.001	0.122
History of depression	1615 (53.9%)	2816 (43.9%)	81.83	<0.001	0.093
History of anxiety	1407 (47.0%)	2493 (38.9%)	56.14	<0.001	0.077
History of schizophrenia	570 (19.1%)	712 (11.1%)	109.04	<0.0010	0.108
History of bipolar or manic depression	399 (13.4%)	564 (8.8%)	45.87	<0.001	0.070
History of drug-induced psychosis	282 (9.4%)	325 (5.1%)	64.18	<0.001	0.083
History of PTSD or other trauma^a^	253 (8.5%)	487 (7.6%)	2.15	0.143	0.015
Experienced mental health symptoms in past month	943 (31.6%)	1242 (19.5%)	168.43	<0.001	0.134
Psychotic symptoms in the past month	418 (14.0%)	482 (7.6%)	97.95	<0.001	0.102
Serious mental illness^b^	905 (30.2%)	1251 (19.5%)	132.31	<0.001	0.118
Common mental disorder^c^	1761 (58.7%)	3130 (48.7%)	81.23	<0.001	0.093
Mental health referral recommended	1447 (47.5%)	2461 (38.1%)	76.23	<0.001	0.090
Mental health referral made	1920 (63.1%)	3344 (51.7%)	107.58	<0.001	0.106
Suicide and self-harm variables					
Ever engaged in self-harm	592 (19.9%)	734 (11.5%)	116.99	<0.001	0.112
Ever attempted suicide	475 (16.0%)	653 (10.2%)	62.92	<0.001	0.082
Any suicidal ideation in past week	292 (9.8%)	384 (6.0%)	43.21	<0.001	0.068
Any self-harm in past 12 months	265 (8.9%)	340 (5.3%)	42.52	<0.001	0.067
Any suicide attempts in past 12 months	188 (6.3%)	280 (4.4%)	15.69	<0.001	0.041
Any self-harm or suicide attempts in past 12 months	323 (10.8%)	467 (7.3%)	32.41	<0.001	0.059
RIT referral recommended	290 (9.5%)	436 (6.7%)	22.69	<0.001	0.049
RIT referral made	385 (12.6%)	624 (9.7%)	19.54	<0.001	0.045

*Note.* Degrees of freedom = 1. Totals ranged from 9411 to 9508 due to missing data.

a This variable was coded from ‘Other mental illness’ prompt.^b^Includes individuals with a history of schizophrenia, psychosis, and/or bipolar disorder. ^c^Includes individuals with a history of depression and/or anxiety.

First Nations prison entrants showed higher prevalence of all mental health variables, apart from having a history of PTSD or other trauma which was not statistically significantly different (8.5% vs 7.6%). The largest effects were observed for ‘any’ mental health condition being reported (70.5% vs 58.0%), history of schizophrenia (19.1% vs 11.1%), experiencing mental health symptoms in the past month (31.6% vs 19.5%), having history of a serious mental illness (30.2% vs 19.5%), and having a mental health referral made (63.1% vs 51.7%). Furthermore, First Nations prison entrants had higher prevalence for all recorded self-harm and suicide risk variables. Variables showing the largest differences included ever having engaged in self-harm (19.9% vs 11.5%), ever attempting suicide (16.0% vs 10.2%) and any self-harm in the past 12 months (8.9% vs 5.3%).

### Combined gender and First Nations identity comparison

Results of further stratification of the study population by both gender and First Nations identity are presented in Table 4. Although First Nations men were more likely to have a history of anxiety (43.8%) compared to non-First Nations men (36.0%), the same statistically significant difference was not found for women (63.2% vs 58.8%). Among women, the algorithm recommended mental health referrals for similar proportions of First Nations women (63.4%) and non-First Nations women (58.1%), though a referral was made for a greater proportion of First Nations women (79.3% vs 70.6%).

**Table 4.** First Nations and non-First Nations people, stratified by gender.

Variable	Men					Women				
	First Nations	Non-First Nations	χ^2^	*p*-value	ϕ	First Nations	Non-First Nations	χ^2^	*p*-value	ϕ
	*n* = 2532	*n* = 5638				*n* = 513	*n* = 823			
Demographic characteristics										
Under 30 years	1140 (45.0%)	1827 (32.4%)	120.30	<0.001	0.121	230 (44.8%)	316 (38.4%)	5.420	0.020	0.064
First time in custody	400 (16.0%)	1714 (30.6%)	190.62	<0.001	–0.153	104 (20.5%)	315 (38.5%)	46.68	<0.001	–0.188
Mental health variables										
Any mental health condition reported	1697 (68.0%)	3110 (55.5%)	111.99	<0.001	0.118	420 (83.0%)	607 (74.1%)	14.18	<0.001	0.103
History of depression	1254 (50.34%)	2303 (41.2%)	58.95	<0.001	0.085	361 (71.3%)	513 (62.7%)	10.38	0.001	0.089
History of anxiety	1088 (43.8%)	2012 (36.0%)	44.26	<0.001	0.074	319 (63.2%)	481 (58.8%)	2.49	0.115	0.043
History of schizophrenia	475 (19.1%)	632 (11.3%)	88.13	<0.001	0.104	95 (18.8%)	80 (9.8%)	22.17	<0.001	0.130
History of bipolar or manic depression	313 (12.6%)	462 (8.3%)	37.12	<0.001	0.068	86 (17.1%)	102 (12.5%)	5.42	0.020	0.064
History of drug-induced psychosis	247 (9.9%)	298 (5.3%)	58.02	<0.001	0.085	35 (6.9%)	27 (3.3%)	9.20	0.002	0.083
History of PTSD or other trauma^a^	180 (7.3%)	381 (6.8%)	0.505	0.477	0.008	73 (14.5%)	106 (13.0%)	.615	0.433	0.022
Experienced mental health symptoms in past month	763 (30.8%)	1010 (18.1%)	149.08	<0.001	0.136	180 (36.1%)	212 (26.2%)	14.32	<0.001	0.105
Psychotic symptoms in the past month	352 (14.2%)	414 (7.4%)	90.98	<0.001	0.106	66 (13.2%)	68 (8.4%)	7.80	0.005	0.077
Serious mental illness^b^	745 (29.9%)	1081 (19.3%)	110.36	<0.001	0.117	160 (31.6%)	170 (20.8%)	19.74	<0.001	0.122
Common mental disorder^c^	1382 (55.4%)	2586 (46.2%)	63.12	<0.001	0.088	379 (74.9%)	562 (68.6%)	6.00	0.014	0.067
Mental health referral recommended	1122 (44.3%)	1983 (35.2%)	61.96	<0.001	0.087	325 (63.4%)	478 (58.1%)	3.66	.056	0.052
Mental health referral made	1513 (59.8%)	2763 (49.0%)	81.26	<0.001	0.100	407 (79.3%)	581 (70.6%)	12.54	<0.001	0.097
Suicide and self-harm variables										
Ever engaged in self-harm	450 (18.2%)	533 (9.6%)	118.18	<0.001	0.121	142 (28.2%)	201 (24.7%)	1.96	0.162	0.039
Ever attempted suicide	377 (15.2%)	574 (10.3%)	49.581	<0.001	0.078	98 (19.5%)	106 (13.1%)	9.74	.002	0.086
Any suicidal ideation in past week	227 (9.2%)	320 (5.7%)	31.42	<0.001	0.062	65 (12.9%)	64 (7.9%)	8.98	0.003	0.083
Any self-harm in past 12 months	195 (7.9%)	248 (4.4%)	38.35	<0.001	0.069	70 (13.9%)	92 (11.3%)	1.90	0.169	0.038
Any suicide attempts in past 12 months	149 (6.0%)	234 (4.2%)	12.35	<0.001	0.039	39 (7.7%)	46 (5.7%)	2.22	0.136	0.041
Any self-harm or suicide attempts in past 12 months	248 (10.0%)	363 (6.5%)	29.65	<0.001	0.061	75 (14.9%)	104 (12.8%)	1.14	0.285	0.029
RIT referral recommended	222 (8.8%)	356 (6.3%)	16.04	<0.001	0.044	68 (13.3%)	80 (9.7%)	4.01	0.045	0.055
RIT referral made	292 (11.5%)	500 (8.9%)	14.21	<0.001	0.042	93 (18.1%)	124 (15.1%)	2.18	0.140	0.040

*Note.* Degrees of freedom = 1. Totals ranged from 9411 to 9508 due to missing data.

a This variable was coded from ‘Other mental illness’ prompt.^b^Includes individuals with a history of schizophrenia, psychosis, and/or bipolar disorder. ^c^Includes individuals with a history of depression and/or anxiety.

Similar to the previously reported pattern, First Nations men had significantly higher prevalence of all self-harm and suicide risk variables compared to non-First Nations men. However, the same was not true for women across the board. Specifically, First Nations and non-First Nations women did not differ on whether they had ever engaged in self-harm (28.7% vs 24.7%), whether they had self-harmed in the past 12 months (13.9% vs 11.3%), whether they had a suicide attempt in the past 12 months (7.7% vs 5.7%), or whether a RIT referral was made (18.1% vs 15.1%).

## Discussion

In a large total population study of people entering public prisons in NSW over a 12-month period, almost two-thirds reported having a mental health condition, just under a quarter reported a serious mental illness, and just under a quarter had experienced recent symptoms of a mental health condition during the month prior to prison entry. Women entering prison had higher prevalence of most mental health diagnoses and higher prevalence of previous self-harm and suicidal ideation/behaviour. First Nations people also reported higher prevalence of most mental health problems as well as self-harm/suicide risk, compared to non-First Nations individuals. In response to such high proportions of people with mental health need and risk, over half of prison entrants were referred to mental health services in prison and one in ten were referred to an in-prison team established to manage risk of self-harm and suicide; all such referral rates being higher for women and First Nations people. These results confirm the enormous burden of mental health problems and self-harm/suicide risk among people in prison, particularly at the point of entry into prison, and particularly among subgroups such as women and First Nations people. There is first a clear need for investment in integrated interventions to prevent those with significant mental health needs from entering prison, including via court diversion initiatives (e.g. Gaskin et al., 2024; Marr et al., 2024; Soon et al., 2024) and community mental health programmes. In addition, adequately resourced prison-based mental health services are required to meet the needs of prison entrants.

### Main findings

Limited comparable work exists examining the prevalence of lifetime mental illness in Australian prison entrants. One Australian study from 2014 examined psychiatric conditions in male prison entrants as identified by a modified risk assessment tool (Schilders and Ogloff, 2014). They found that 39% of men entering a Victorian prison had a lifetime psychiatric condition (including psychotic disorders, mood disorders, and anxiety disorders), compared to the 62% of our cohort who self-reported a history of any mental health condition.

Other prison surveys are often limited by response rates and the potential for sampling bias, compared to our total population approach. For example, the median-reported response rate in studies included in the Fazel and Seewald (2012) meta-analysis was 81%. Rates of mental illness in these studies may be underestimating the prevalence of mental illness given that those decline to participate or without capacity to consent to participation are more likely to be experiencing symptoms of mental illness. Our main findings suggest high rates of mental health conditions, symptoms, and self-harm or suicide risk of prison entrants, in line with recent meta-analytic work in Australian prison contexts (e.g. Yee et al., 2024).

Self-reported history of self-harm or suicide attempts was also high among those in our study population: 14.2% reporting a history of self-harm and 12.1% a previous suicide attempt. However, these rates of lifetime self-harm and suicide attempts are lower than reported in other national studies of people in prison. For example, Browne et al. (2022a) found that 32.5% of a sample of prison entrants in NSW reported a previous suicide attempt and Larney et al. (2012) surveyed adults in NSW prisons and found that 20.5% of respondents reported the a previous suicide attempt. This study’s findings may be an underestimate of prior self-harm or suicide attempts given the questions are asked by health staff rather than independent researchers. Still, these prevalence rates are higher than those found in the community: 4.9% of Australians have reported a history of self-harm and 8.7% reported a previous suicide attempt (Australian Bureau of Statistics, 2023). In addition, poor mental health is a key risk factor for self-harm/suicide behaviours, as evidenced by previous research in prison settings (e.g. Favril et al., 2020; Schilders and Ogloff, 2014). Indeed, the high rates of past and/or current mental health conditions, symptoms of mental illness and self-harm/suicide behaviour and ideation identified at screening resulted in around every one in ten prison entrants being referred to the RIT for further assessment and/or management of risk of suicide/self-harm. Such a large number of referrals for risk assessment and management clearly requires significant resourcing, though is similar to prevalence of self-harm/suicide risk found in other prison screening studies (e.g. VISCI; Hausam et al., 2024).

Comparisons across studies are limited by differences in methodology, including whether or not diagnostic thresholds are used, whether current or lifetime prevalence are reported, and what approach to eliciting symptoms or previous psychiatric history is employed. This study aimed to establish the prevalence of lifetime psychiatric conditions and current symptoms based on self-report captured on health screening. It is thus likely to find higher prevalence when compared to studies that report current illness above diagnostic thresholds based on structured diagnostic interview. However, it is important to note that prison-based mental health services need to respond to mental health problems and self-harm/suicide risks that go beyond that defined by diagnostic thresholds and thus knowing the prevalence for a broader range of needs and risks is vital for the provision of adequate services and interventions.

### Men, women and First Nations people

Our findings suggest that, in general, women entering prison have a higher prevalence of mental health conditions, symptoms of mental illness and indicators of self-harm and suicide risk than men, in line with some (e.g. Tyler et al., 2019), but not all previous research (Fazel and Seewald, 2012). In NSW, Browne et al. (2022b) also found that women in prison reported higher prevalence of most mental illness diagnoses compared to men in prison, and that this discrepancy was only increasing over the years.

In this study, the only exception to the finding that women entering prison had higher prevalence of mental health conditions and risk than men was having a history of drug-induced psychosis (6.8% of men; 4.7% of women). This aligns with previous research using clinical samples (e.g. Caton et al., 2014), though limited comparable work is available for samples of individuals entering prison. There were also two categories of mental health conditions that did not differ between men and women – having a history of schizophrenia (13.7% vs 13.2%) and experiencing psychotic symptoms in the past month (9.5% vs 10.3%). This is in line with general work suggesting similar lifetime prevalence of psychotic disorders for both genders, with higher incidence for men than women (Barajas et al., 2015; Hanlon et al., 2017; Ochoa et al., 2012).

Furthermore, regarding indicators of self-harm and suicide risk, we found that 7.6% of men and 13.5% of women reported any self-harm or suicide attempt within the past year. A similar pattern was observed by Browne et al. (2022), who found that of 341 prison entrants in NSW, 8.7% of men and 8.9% of women reported a suicide attempt in the past 12 months.

These findings highlight that while the numbers of women entering prison are much lower than men, their mental health needs and risks are greater, indicating a high rate of disadvantage among women who become incarcerated. Women in prison often have histories of trauma and complex needs (Bartlett and Hollins, 2018). There is an ongoing need to provide women in prison with greater mental health support to promote positive mental health within prison (e.g. Bright et al., 2022), as well as a need to focus on prevention of incarceration in the first place.

Findings from our study also show that, across the board, First Nations people entering prison had higher prevalence of mental health conditions and histories of self-harm and suicidal behaviour, and this was also mostly true when we stratified results by gender. Overall, we found that 70.5% of First Nations prison entrants had reported any mental health condition, compared to 58.0% of non-First Nations people. This proportion is line with other studies of First Nations people in Australian prisons, many of which have examined men and women separately. For example, one Queensland study found that 72.8% of First Nations men and 86.1% of First Nations women had at least one mental health condition in the preceding 12 months (Heffernan et al., 2012). Another study in Victoria found that 68.9% of men and 76.9% of women had been diagnosed with a mental illness during their lifetime (Ogloff et al., 2017). Descriptively, our study also showed that First Nations women showed the highest prevalence for most variables compared to all other groups.

Given these findings, it is crucial that targeted mental health interventions are implemented to address the disproportionately high proportion of mental health conditions among First Nations prison entrants. Tailored approaches that consider cultural and gender-specific needs are essential to effectively support First Nations people in prison, and particularly First Nations women (Stewart et al., 2021). Studies have shown that culturally appropriate interventions, such as those involving Indigenous-led mental health services, can lead to better health outcomes for First Nations people (Dudgeon et al., 2014). Examples of successful programmes include Queensland’s Indigenous Mental Health Intervention Program (IMHIP, 2018; Dale et al., 2023). The First Nations-led multidisciplinary programme implements a culturally-informed model of care in prison. The programme aims to accurately identify mental health and social and emotional well-being needs (beyond Western conceptualisations), provides culturally supportive care in prison and assists people with the transition from prison back to the community.

Furthermore, the allocation of adequate funding and resources is imperative to ensure that First Nations individuals in the criminal justice system have access to appropriate mental health services in prison. Reducing the number of First Nations people with mental health conditions in prison should be a key priority. This includes diverting individuals with mental illness away from the criminal justice system, providing assertive treatment in the community – particularly for First Nations women, as well as ensuring that mental health care available in the community is culturally informed and safe.

#### Strengths and limitations

This study comprehensively described the prevalence of a range of mental health and self-harm/suicide risk indicators across all entrants to public prisons in NSW over a 1-year period. This approach limits selection bias and thus provides a more accurate profile of people entering prison. With a large, representative study population, we were also able to make comparisons by gender and First Nations identity.

However, our study also had some limitations. The routine screening focused on lifetime history of mental health conditions, meaning that more precise estimates of the timing of disorder relative to prison entry could not be established. Current or recent symptoms of mental health conditions were obtained; however, these screening data are based on self-report in response to structured questions posed by nursing staff employed by the health service responsible for their care while in prison. Increased stress and psychological distress at prison entry could lead to inflated estimates of mental health symptoms but prison entrants may also under-report previous diagnoses, current symptoms of mental illness, or past or current self-harm or suicide experiences. As noted earlier, our findings may indicate some evidence of this – in our study population, 16.0% of First Nations prison entrants reported a history of suicide attempts. Hesitancy to report previous self-harm or suicide attempts at screening may stem from a desire to avoid increased observations and restrictions during their time in prison. Prison entrants also likely have different mental health needs than the broader prison population, thus limiting the generalisability of these findings beyond prison entry. Furthermore, these self-reported data are also not directly analogous to data presenting prevalence of mental illness based on structured diagnostic interviews and the application of diagnostic thresholds. Although this limits comparability of our findings to such studies, it is important to note that such studies are prone to selection and information biases and likely underestimate the true burden of mental health problems and risk among those entering prison.

Our study also focused on mental health conditions, symptoms of mental illness and self-harm/suicidal behaviour, but did not consider a broader range of mental health conditions or co-occurring drug and alcohol use problems. Other large prison screening studies have found that the majority of prison entrants have co-occurring mental health needs and substance use and that such comorbidity may be increasing among prison entrants (Butler et al., 2022). There is clearly a need for prison health services to take a more integrated approach to addressing mental health and substance use needs among people entering custodial settings.

Finally, we presented prevalence of mental health conditions and self-harm/suicide risk indicators among First Nations people. It is critical to recognise that these variables reflect Western conceptualisations of health. The existing screening approach may not be sufficient for identifying and understanding the social and emotional well-being of First Nations people (e.g. Calma et al., 2017; Kendall et al., 2019). In reporting these data, we highlight the need to reduce the number of First Nations people entering custody and further work to improve the health and well-being of First Nations people in contact with the criminal justice system. These ideas are in line with the existing Closing the Gap (n.d.) targets set out by the Australian Government, with priority reforms calling for government institutions to embed cultural safety practices and develop services in partnership with First Nations organisations, communities, and people. Doing so is particularly critical in criminal justice settings.

## Conclusion

Taking a total population approach, the findings of this study highlight the enormous burden of mental health problems and self-harm/suicide risk among people entering prison, higher than has been reported previously in studies using non-screening samples and with smaller sample sizes. Consistent with international and local findings, women entering prison reported a higher prevalence of mental health and self-harm and suicide risk indicators than men, with the exception of psychotic illnesses or symptoms. First Nations people also reported a higher prevalence of nearly all mental health and self-harm/suicide risk indicators compared to non-First Nations people, with largest effects shown for recent experience of mental health symptoms and serious mental illness. These findings establish the scale of mental health need and self-harm/suicide risk among people entering prison and highlight particularly vulnerable groups including women and First Nations people. The level of need and risk presented warrants increased investment in preventive efforts, including by community-based mental health services, mental health diversion, and prison-based mental health services.

## Supplemental Material

sj-docx-1-anp-10.1177_00048674251336031 – Supplemental material for Mental health and self-harm/suicide risk screening at prison entry over 12 months in a total population sample in New South Wales, Australia Supplemental material, sj-docx-1-anp-10.1177_00048674251336031 for Mental health and self-harm/suicide risk screening at prison entry over 12 months in a total population sample in New South Wales, Australia by Carey Marr, Christie Browne, Dylan Ngui, Reem Zeki, Emma Woods and Kimberlie Dean in Australian & New Zealand Journal of Psychiatry
