# Supplementary material for: Mental health and self-harm/suicide risk screening at prison entry over 12 months in a total population sample in New South Wales, Australia
Source: Aust N Z J Psychiatry. 2025 Apr 28;59(7):629–39. doi: 10.1177/00048674251336031 (PMC12181648; doi:10.1177/00048674251336031)
Supplement: sj-docx-1-anp-10.1177_00048674251336031 – Supplemental material for Mental health and self-harm/suicide risk screening at prison entry over 12 months in a total population sample in New South Wales, Australia [file sj-docx-1-anp-10.1177_00048674251336031.docx]

**Reception Screening Assessment – Relevant questions**

**Mental Health History**

1. Have you ever been told or think you had Depression?
   1. If yes, have you had symptoms of this in the past months?
2. Have you ever been told or think you had Anxiety?
   1. If yes, have you had symptoms of this in the past months?
3. Have you ever been told or think you had Schizophrenia?
   1. If yes, have you had symptoms of this in the past months?
4. Have you ever been told or think you had Bipolar disorder?
   1. If yes, have you had symptoms of this in the past months?
5. Have you ever been told or think you had Drug-Induced Psychosis?
   1. If yes, have you had symptoms of this in the past months?
6. Have you ever been told or think you had Other Mental Health Problems such as ADHD, Autism, Asperger’s, PTSD, etc.?
   1. If yes, please specify.
   2. If yes, have you had symptoms of this in the past months?

**Mental Health Symptoms**

1. Have you ever heard things that other people couldn’t, such as noises, or the voices of people whispering or talking; OR had visions or saw things that other people couldn’t see?
   1. If yes, has this happened in the past month?
2. Have you ever felt that you were especially important in some way, or had special powers to do things that others could not do?
   1. If yes, has this happened in the past month?
3. Have you ever felt as if your thoughts were being broadcast out loud or that other people could actually hear what you were thinking; or believed that someone could read your mind?
   1. If yes, has this happened in the past month?
4. Have you ever felt depressed or down for most of the day, nearly every day for at least two weeks?
   1. If yes, has this happened in the past month?
5. Have you ever lost interest or pleasure in things you usually enjoyed, nearly every day for at least two weeks?
   1. If yes, has this happened in the past month?
6. Have you ever experienced a period of time when you were feeling so good, “high”, excited, or hyper that other people thought you were not your normal self?
   1. If yes, has this happened in the past month?
7. Have you ever found it hard to do your usually work, take care of things at home, or get along with other people for a sustained period?
   1. If yes, has this happened in the past month?
8. Have you ever had difficulty thinking or concentrating, or making decisions about everyday things?
   1. If yes, has this happened in the past month?
9. Have you ever experienced a change in sleep (trouble falling asleep, waking frequently, trouble staying asleep, waking too early, sleeping too much, needing less sleep than usual and still feeling rested)?
   1. If yes, has this happened in the past month?

**Suicide/Self-Harm Risk Assessment**

1. Have you ever tried to hurt yourself?
   1. If yes, when was the last time you hurt yourself?
   2. How did you hurt yourself?
2. Have you ever tried to end your life?
   1. If yes, when was the last time you tried to end your life?
   2. How did you try to end your life?
3. In the past week, have you been thinking that you might be better off dead?
4. In the past week, have you had any thoughts that life is not worth living?
5. In the past week, have you had thoughts about hurting or killing yourself?
6. In the past week, have you had thoughts about how you might end your life?
7. Is this your first time in prison?
8. Is there anything causing you concern?
   1. If yes, what is it?
9. Do you think you will be able to cope in prison?
